# Supplementary figures and images for: A ‘torn bag mechanism’ of small extracellular vesicle release via limiting membrane rupture of en bloc released amphisomes (amphiectosomes)
Source: eLife. 2025 Feb 7;13:RP95828. doi: 10.7554/eLife.95828 (PMC11805505; doi:10.7554/eLife.95828)

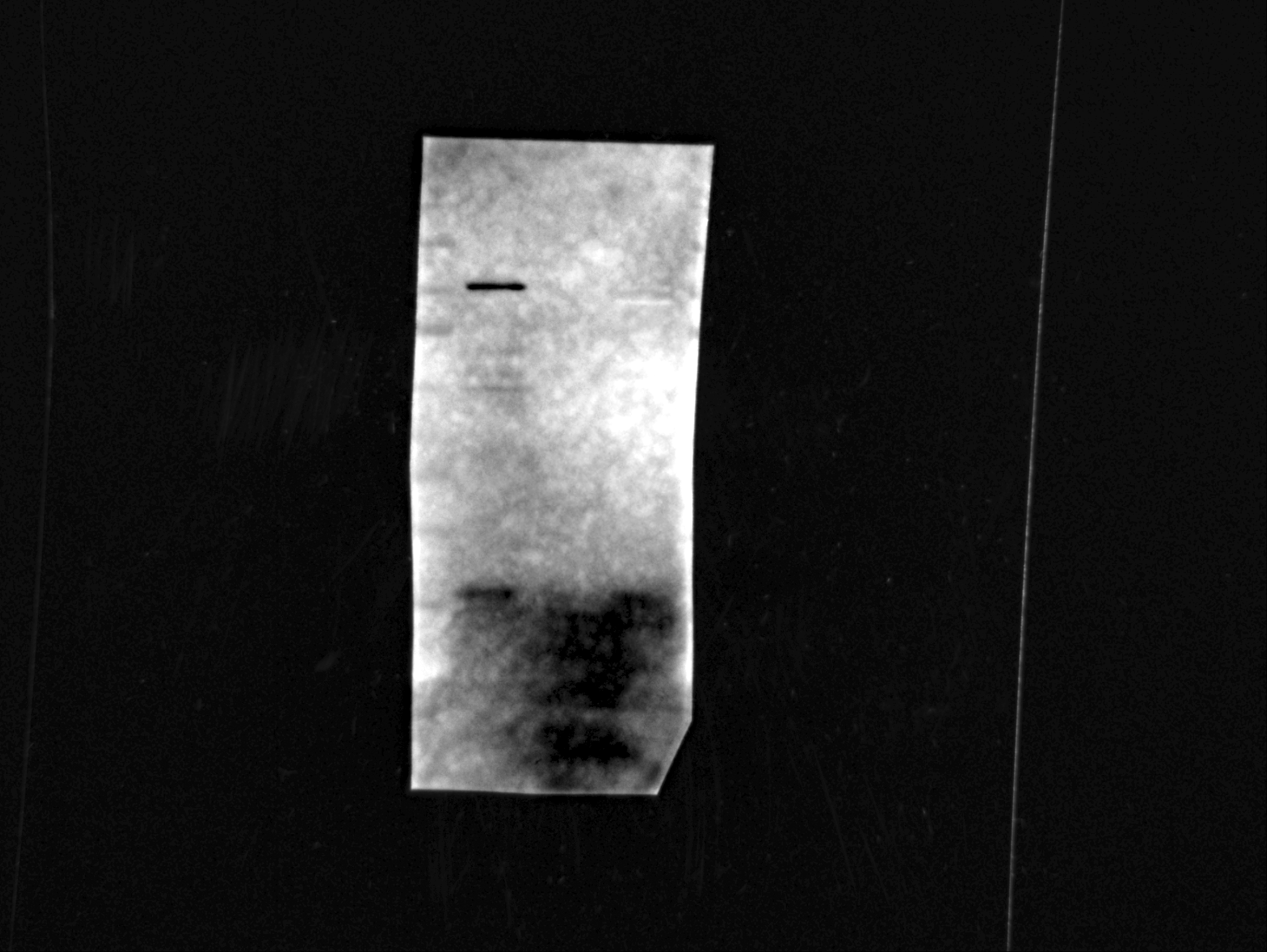

Supplement: Figure 2—figure supplement 4—source data 1. [file elife-95828-fig2-figsupp4-data1.zip › Figure 2-figure supplement 4-source data 1/anti-ALIX Merck SAB4200477.tif]

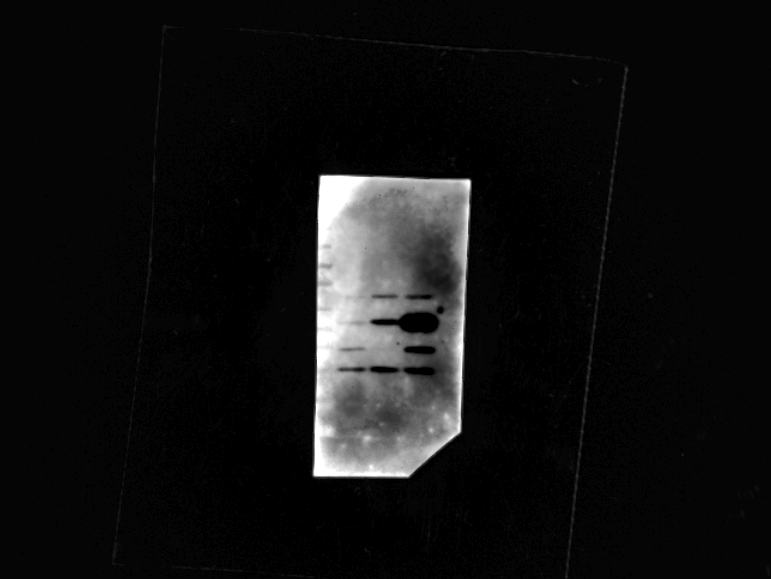

Supplement: Figure 2—figure supplement 4—source data 1. [file elife-95828-fig2-figsupp4-data1.zip › Figure 2-figure supplement 4-source data 1/anti-CD63 Merck SAB2109138.tif]

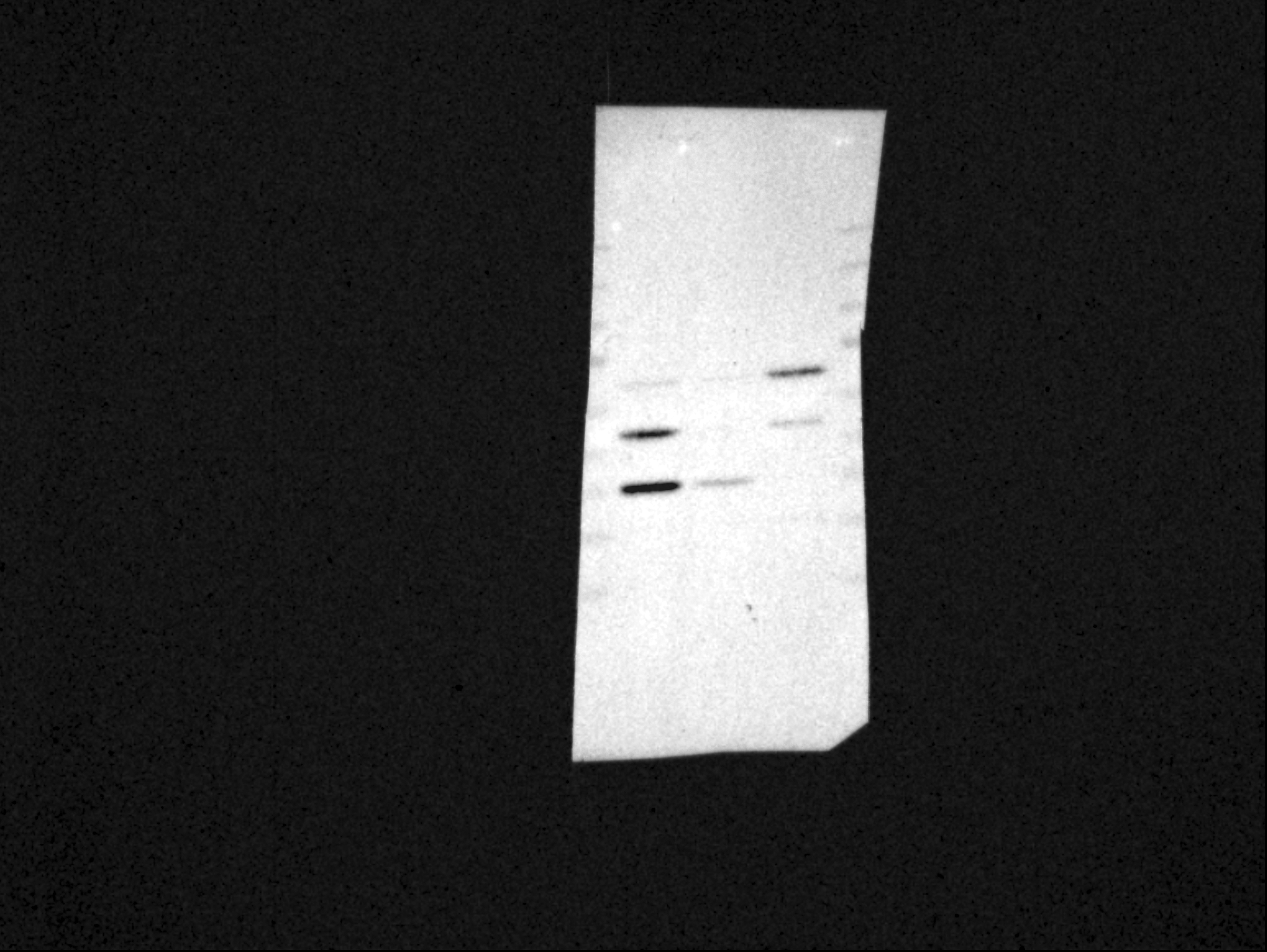

Supplement: Figure 2—figure supplement 4—source data 1. [file elife-95828-fig2-figsupp4-data1.zip › Figure 2-figure supplement 4-source data 1/anti-CD63 Santa Criz, MX-49.129.5.tif]

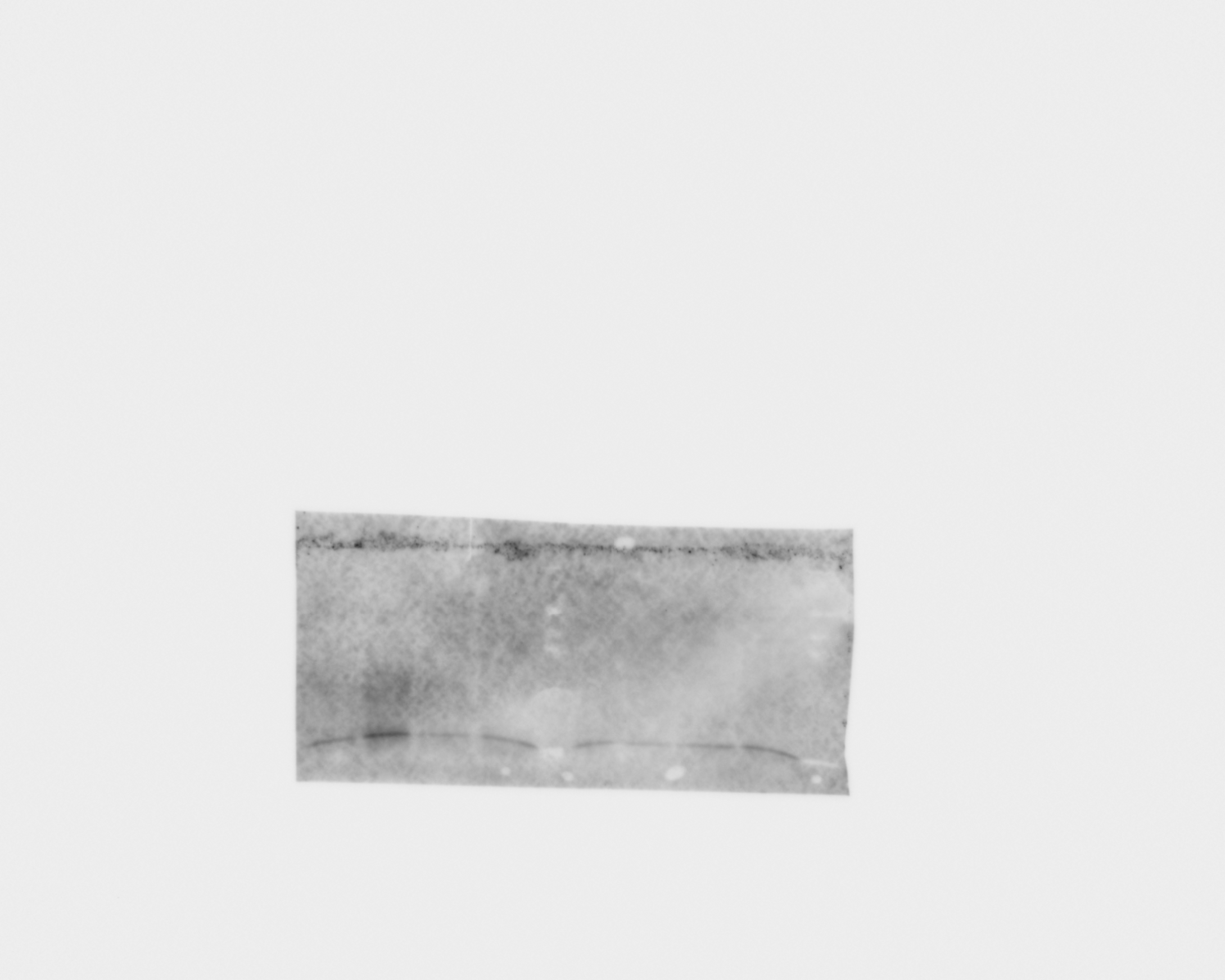

Supplement: Figure 2—figure supplement 4—source data 1. [file elife-95828-fig2-figsupp4-data1.zip › Figure 2-figure supplement 4-source data 1/anti-CD81 Invitrogen MA5-13548.tif]

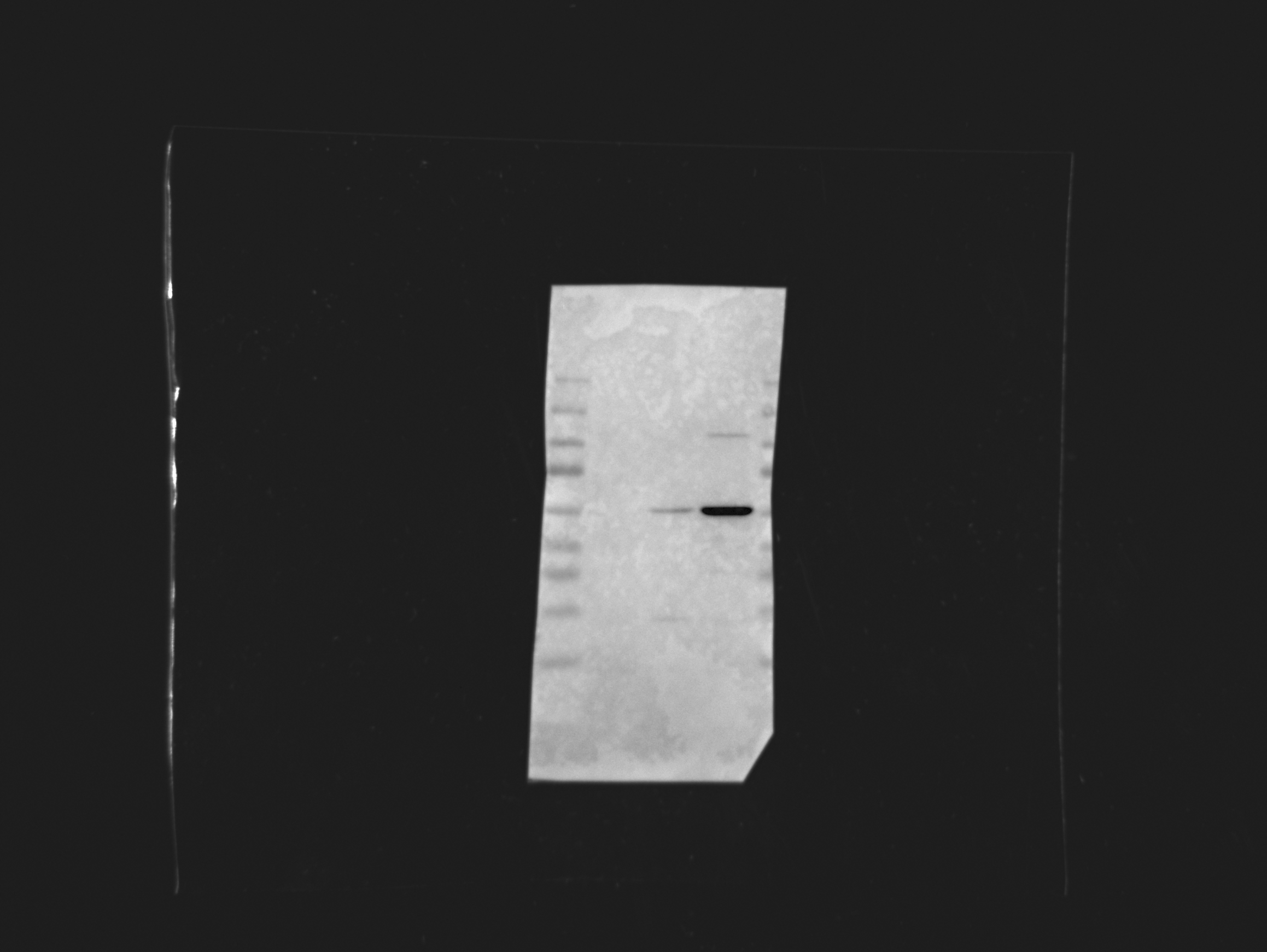

Supplement: Figure 2—figure supplement 4—source data 1. [file elife-95828-fig2-figsupp4-data1.zip › Figure 2-figure supplement 4-source data 1/anti-CD81 Merck SAB3500454.tif]

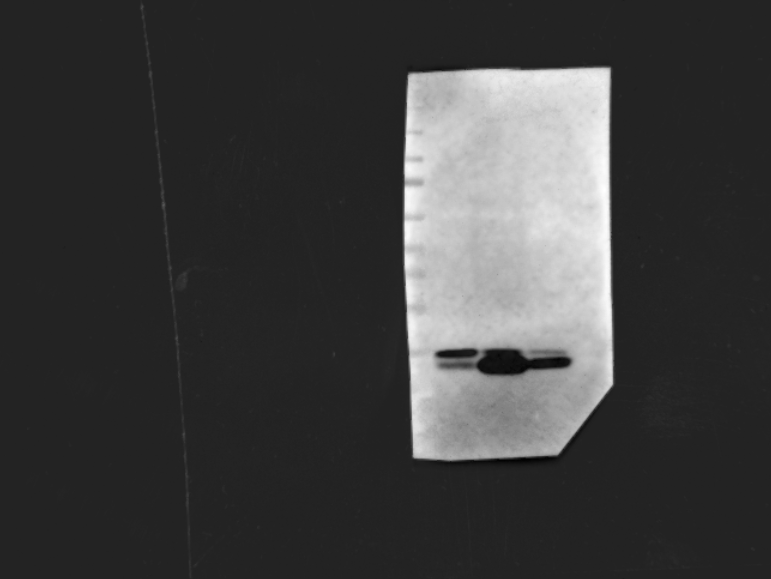

Supplement: Figure 2—figure supplement 4—source data 1. [file elife-95828-fig2-figsupp4-data1.zip › Figure 2-figure supplement 4-source data 1/anti-LC3B_rabbit Sigma ZRB100.tif]

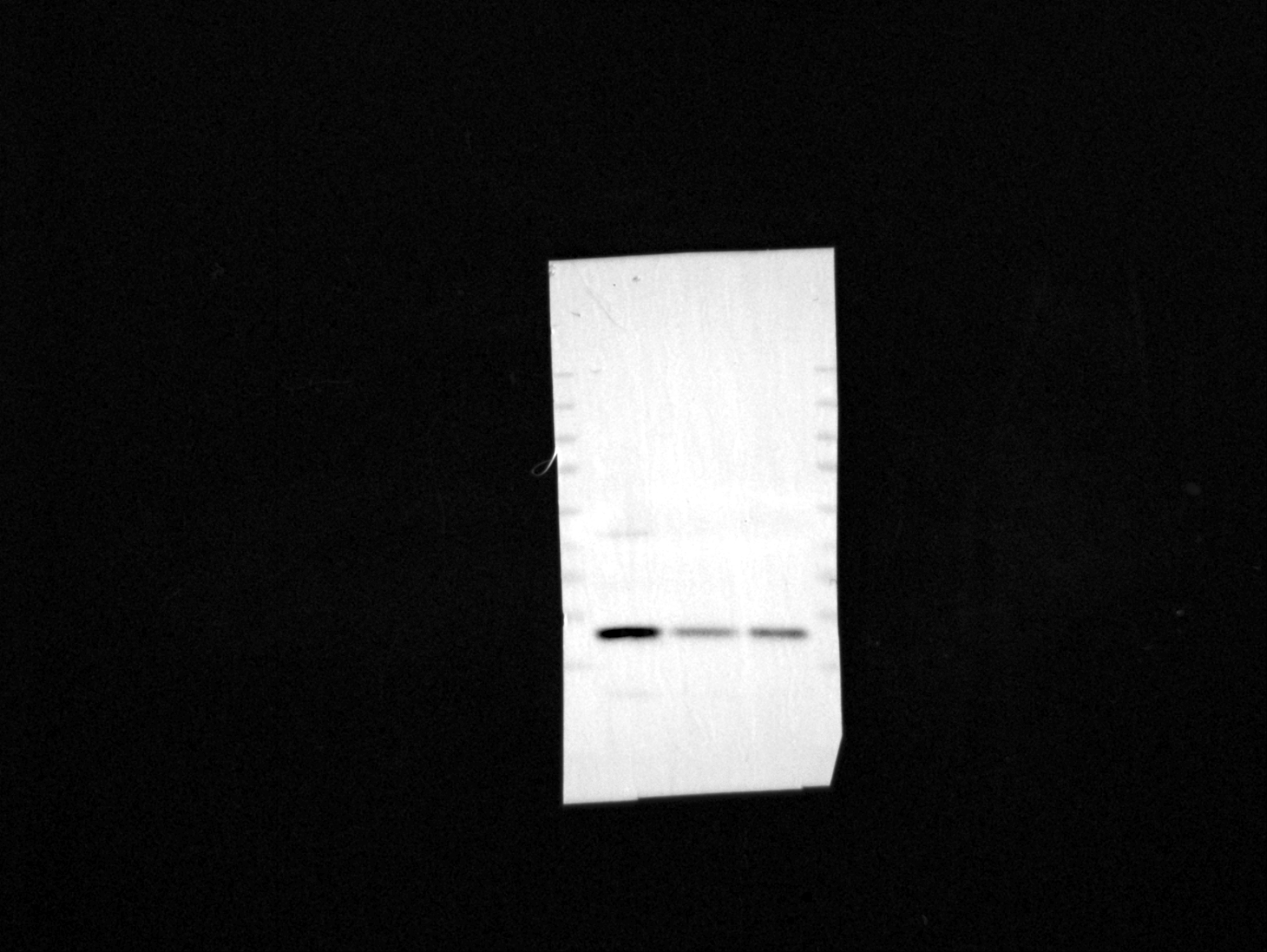

Supplement: Figure 2—figure supplement 4—source data 1. [file elife-95828-fig2-figsupp4-data1.zip › Figure 2-figure supplement 4-source data 1/anti-RAB7_Sigma R8779.tif]

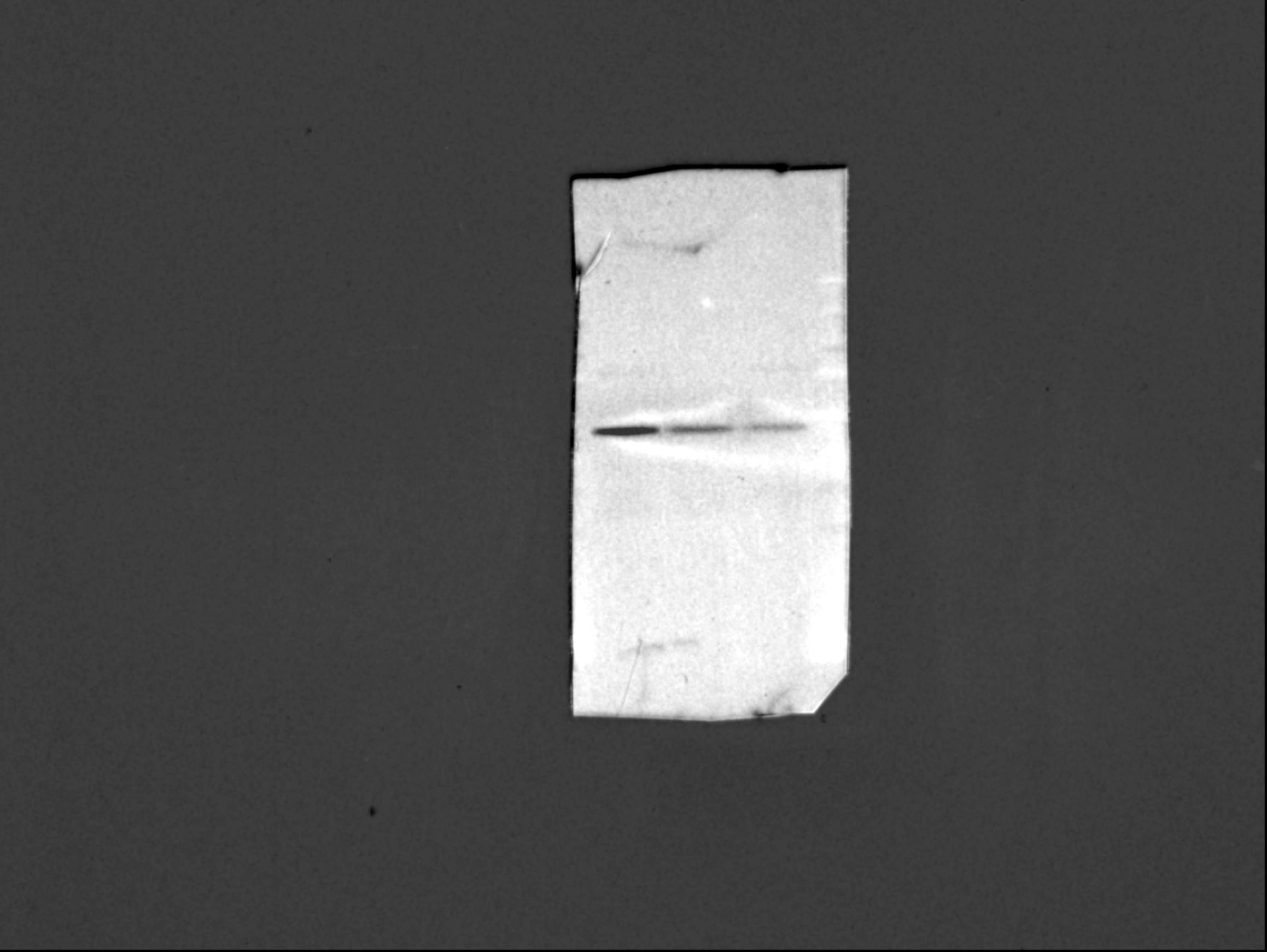

Supplement: Figure 2—figure supplement 4—source data 1. [file elife-95828-fig2-figsupp4-data1.zip › Figure 2-figure supplement 4-source data 1/anti-TSG101 Sigma HPA006161.tif]

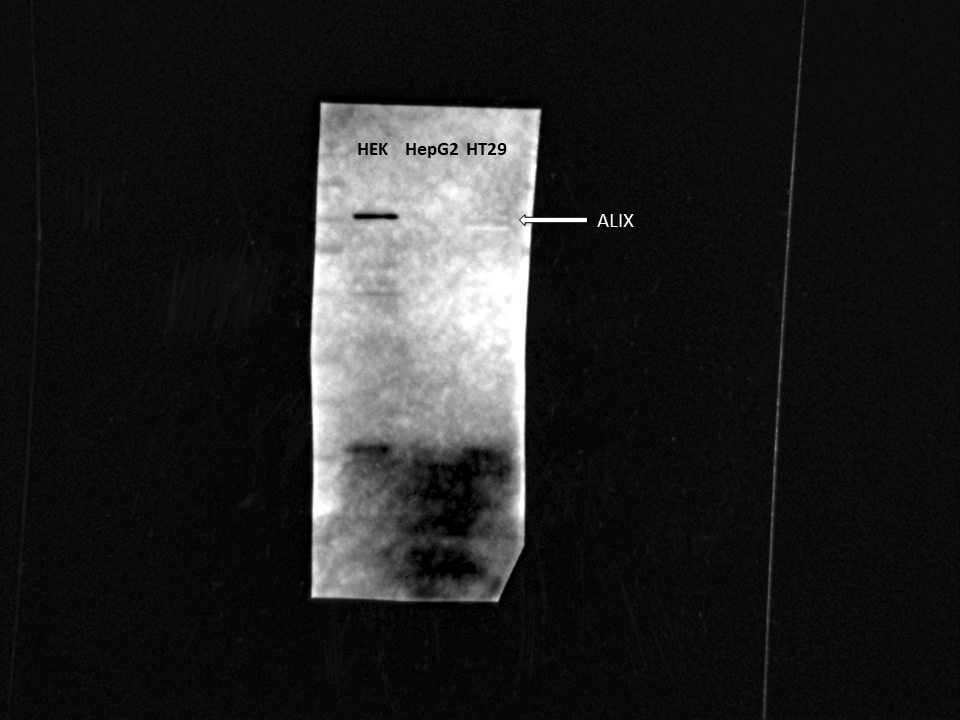

Supplement: Figure 2—figure supplement 4—source data 2. [file elife-95828-fig2-figsupp4-data2.zip › Figure 2-figure supplement 4-source data 2/anti-ALIX Merck SAB4200477.TIF]

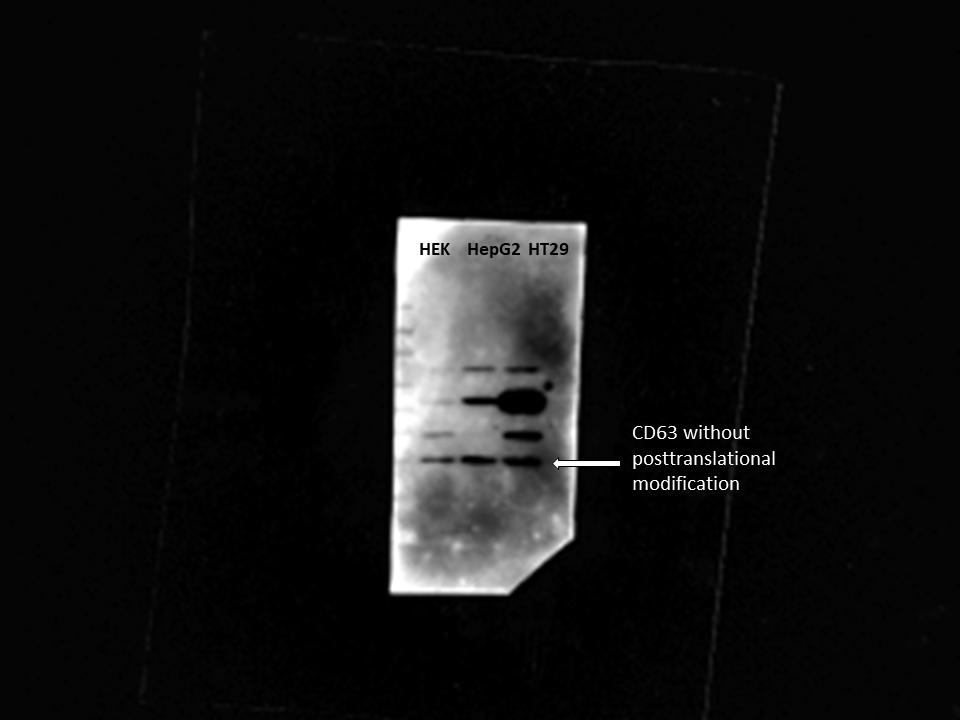

Supplement: Figure 2—figure supplement 4—source data 2. [file elife-95828-fig2-figsupp4-data2.zip › Figure 2-figure supplement 4-source data 2/anti-CD63 Merck SAB2109138.TIF]

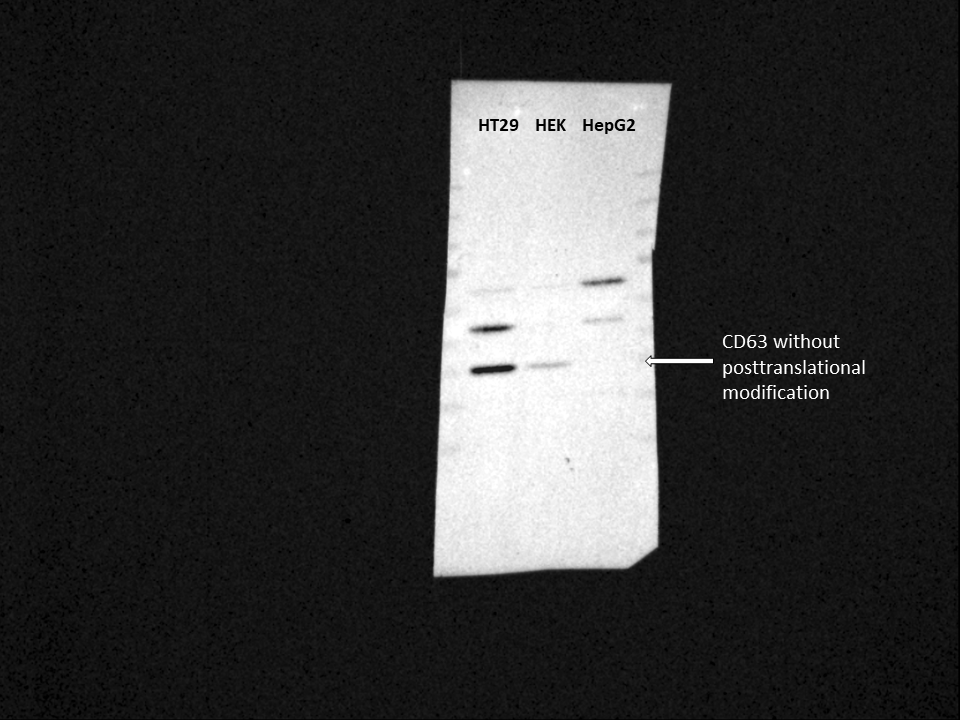

Supplement: Figure 2—figure supplement 4—source data 2. [file elife-95828-fig2-figsupp4-data2.zip › Figure 2-figure supplement 4-source data 2/anti-CD63 Santa Cruz MX 49.129.5.TIF]

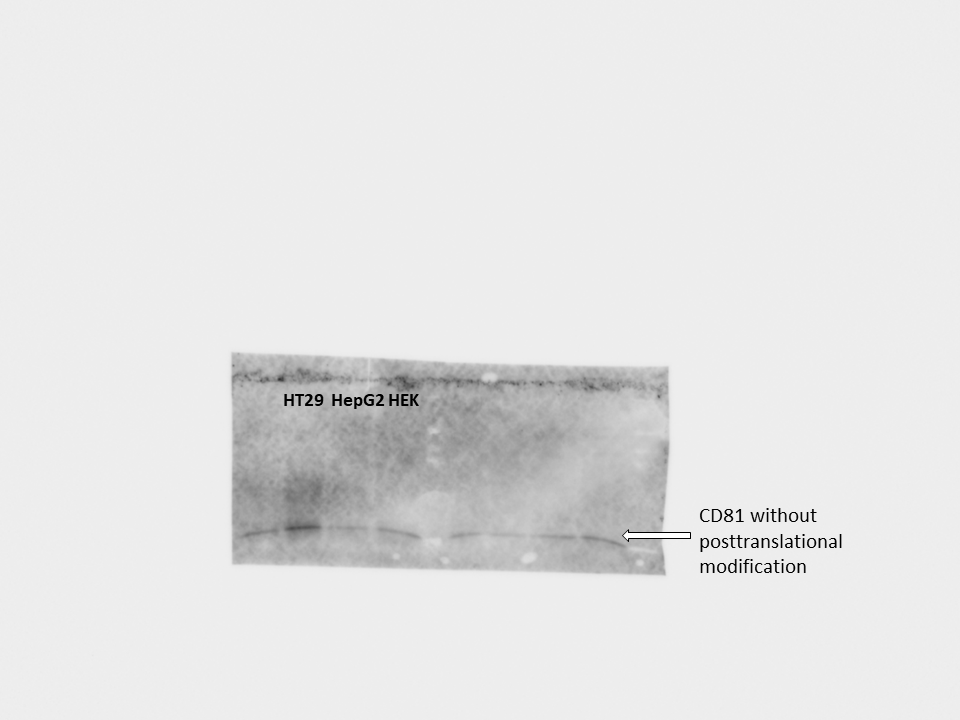

Supplement: Figure 2—figure supplement 4—source data 2. [file elife-95828-fig2-figsupp4-data2.zip › Figure 2-figure supplement 4-source data 2/anti-CD81 Invitrogen MA5-13548.TIF]

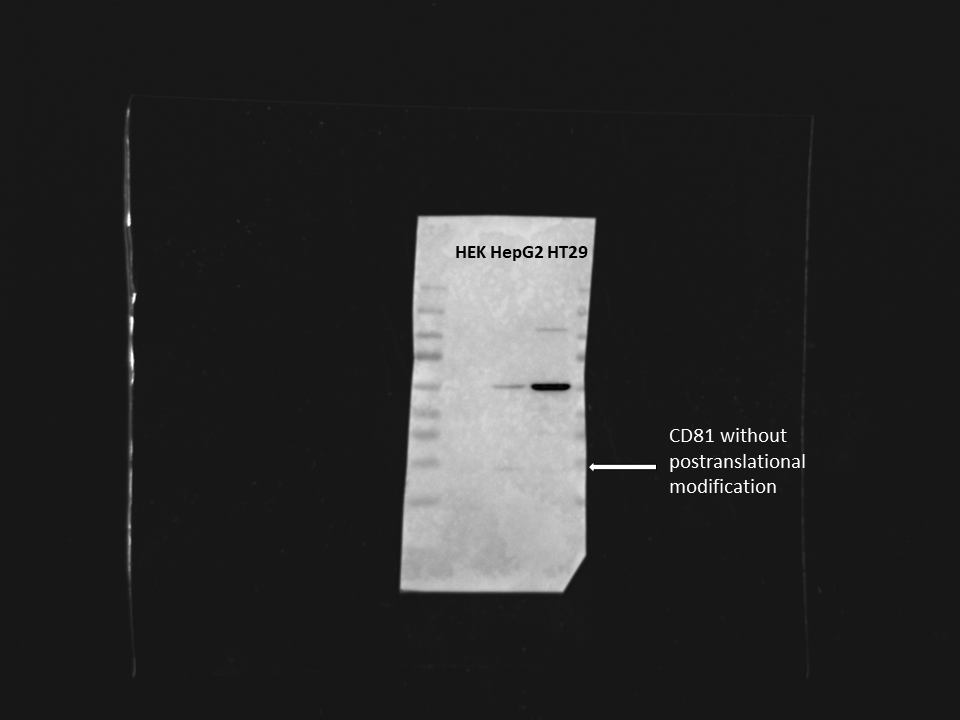

Supplement: Figure 2—figure supplement 4—source data 2. [file elife-95828-fig2-figsupp4-data2.zip › Figure 2-figure supplement 4-source data 2/anti-CD81 Merck SAB3500454.TIF]

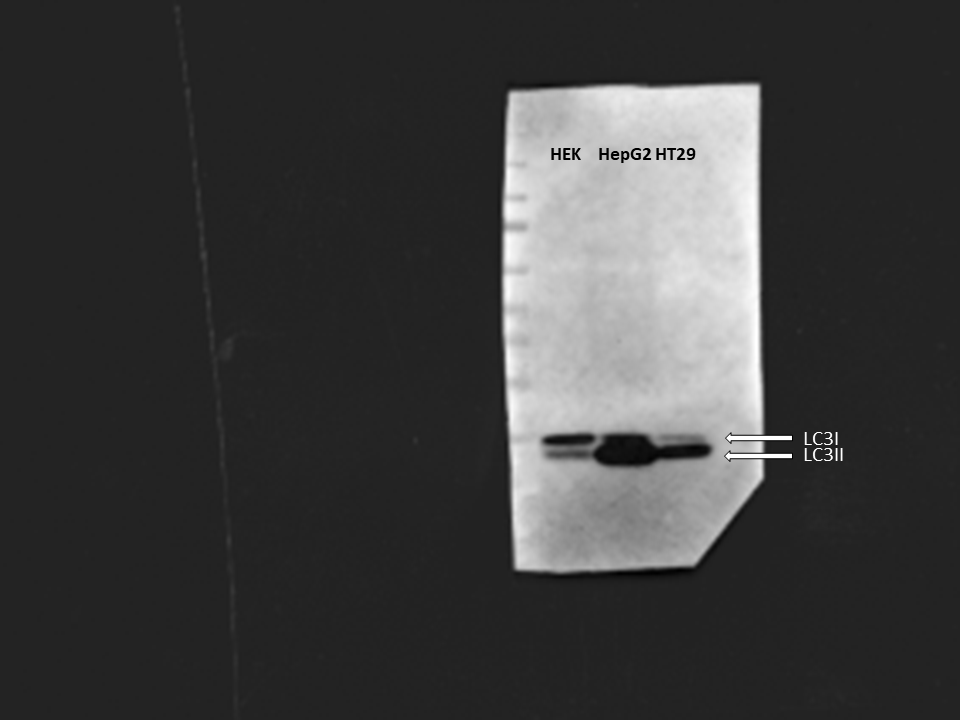

Supplement: Figure 2—figure supplement 4—source data 2. [file elife-95828-fig2-figsupp4-data2.zip › Figure 2-figure supplement 4-source data 2/anti-LC3B Sigma ZRB100.TIF]

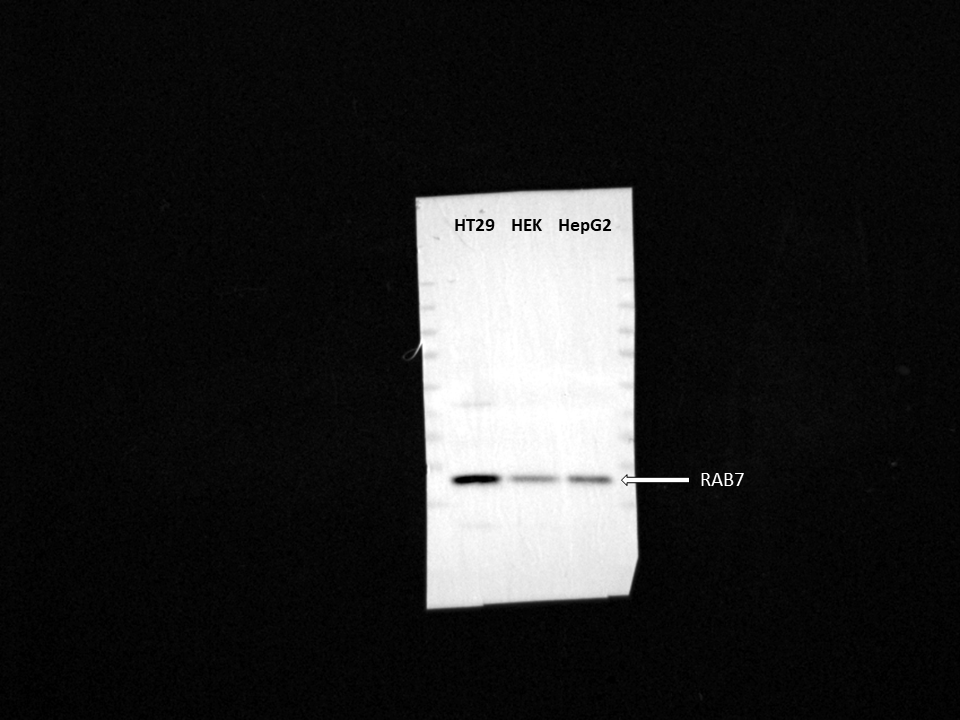

Supplement: Figure 2—figure supplement 4—source data 2. [file elife-95828-fig2-figsupp4-data2.zip › Figure 2-figure supplement 4-source data 2/anti-RAB7 Sigma R8779.TIF]

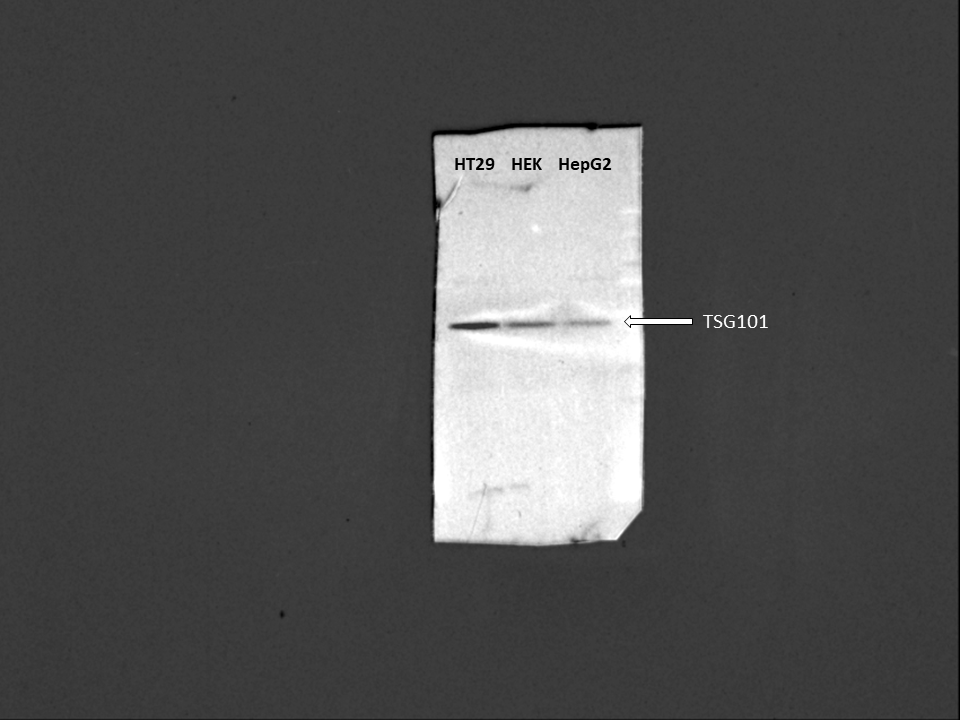

Supplement: Figure 2—figure supplement 4—source data 2. [file elife-95828-fig2-figsupp4-data2.zip › Figure 2-figure supplement 4-source data 2/anti-TSG101 Sigma HPA006161.TIF]

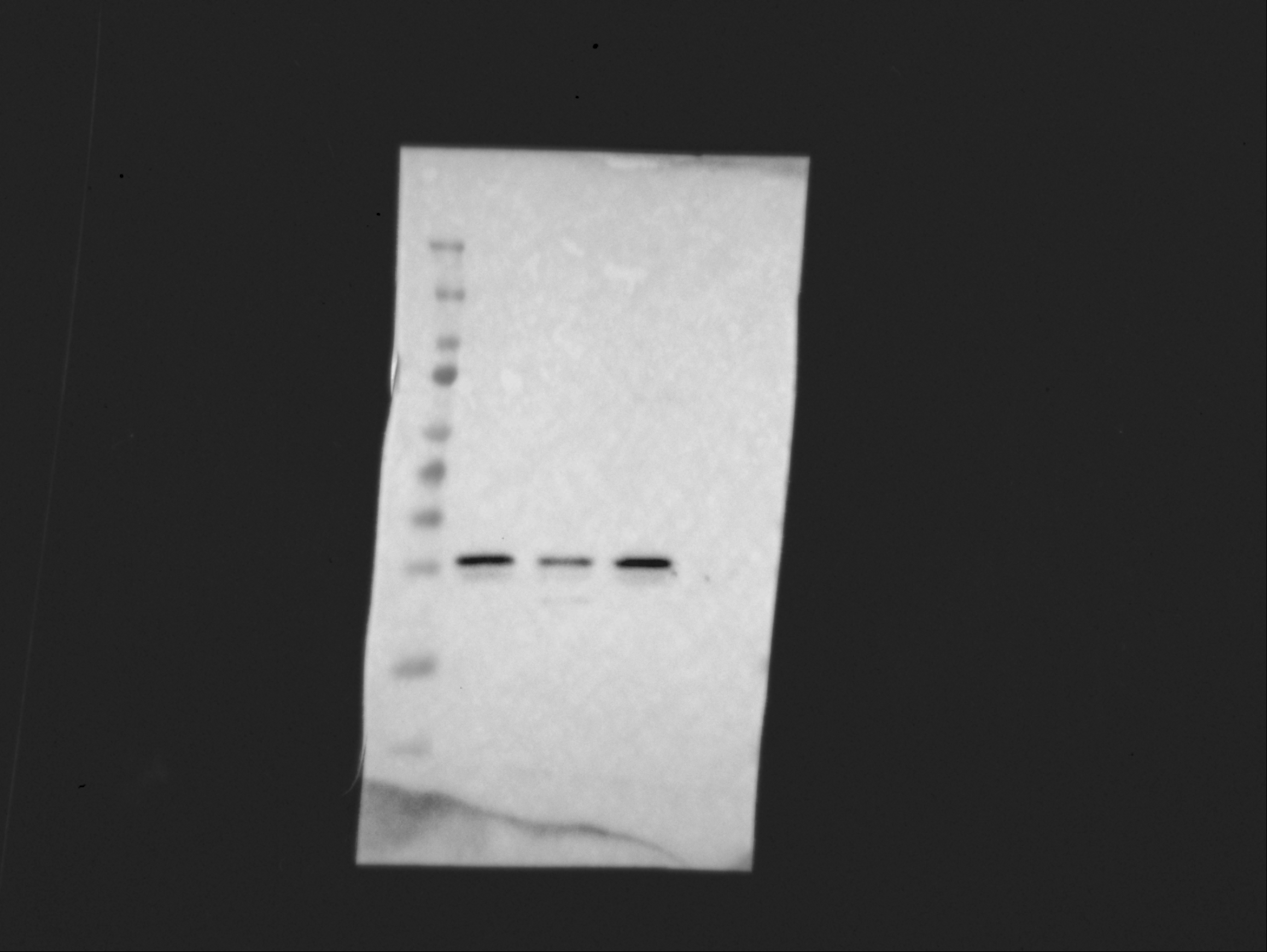

Supplement: Figure 2—figure supplement 5—source data 1. [file elife-95828-fig2-figsupp5-data1.zip › Figure 2-figure supplement 5-source data 1/anti-GFP.tif]

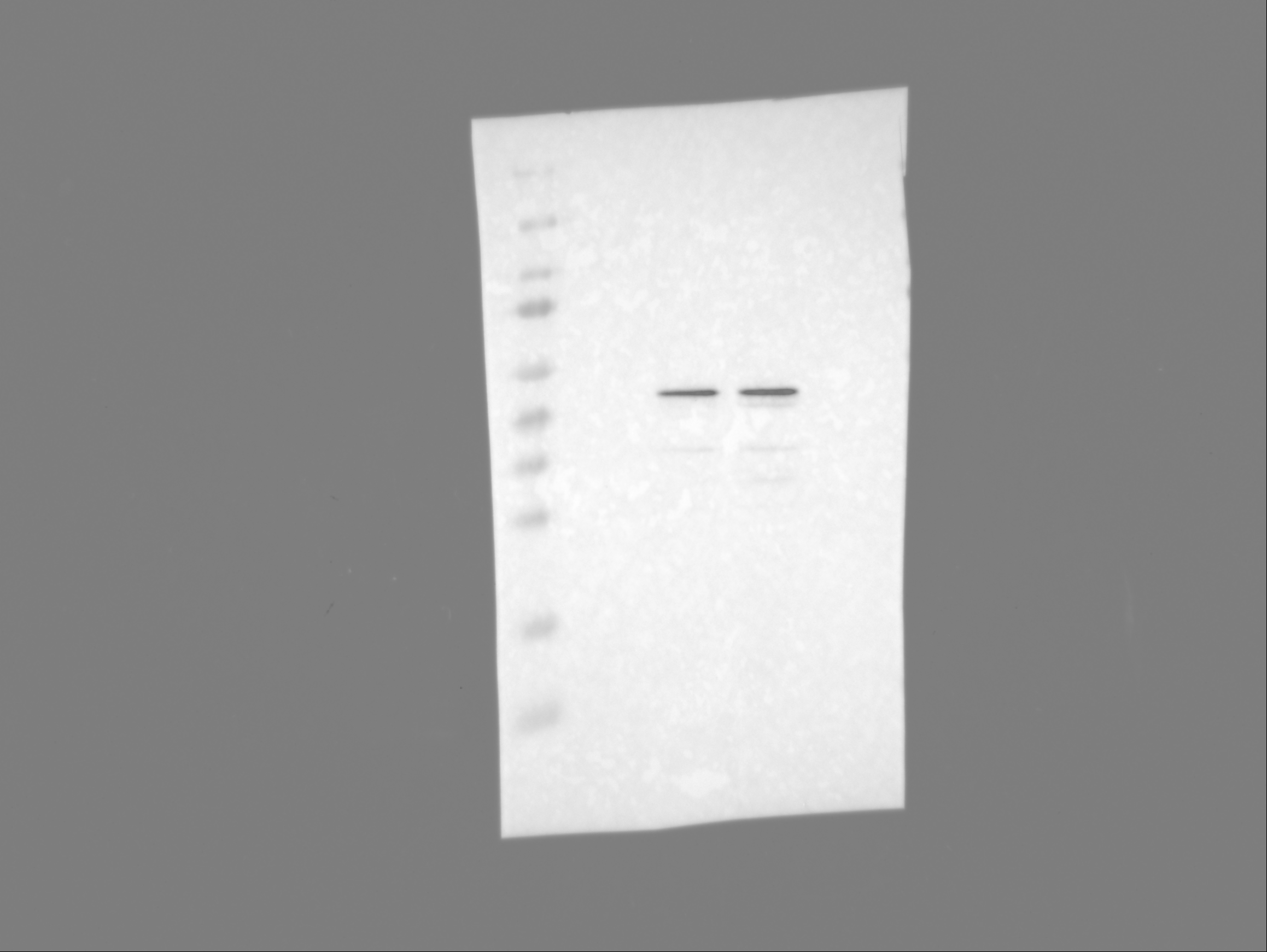

Supplement: Figure 2—figure supplement 5—source data 1. [file elife-95828-fig2-figsupp5-data1.zip › Figure 2-figure supplement 5-source data 1/anti-LC3A.tif]

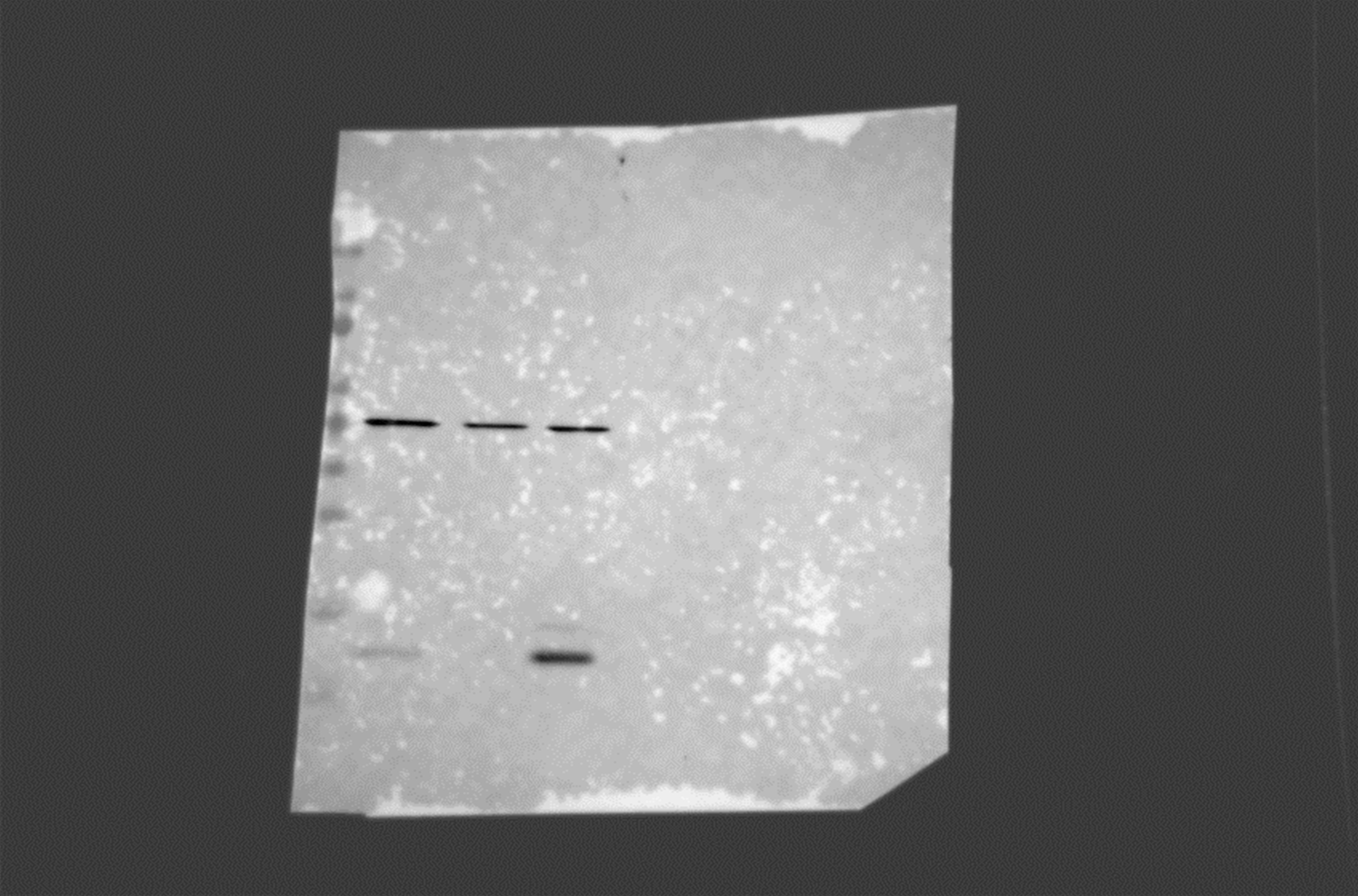

Supplement: Figure 2—figure supplement 5—source data 1. [file elife-95828-fig2-figsupp5-data1.zip › Figure 2-figure supplement 5-source data 1/anti-LC3B.tif]

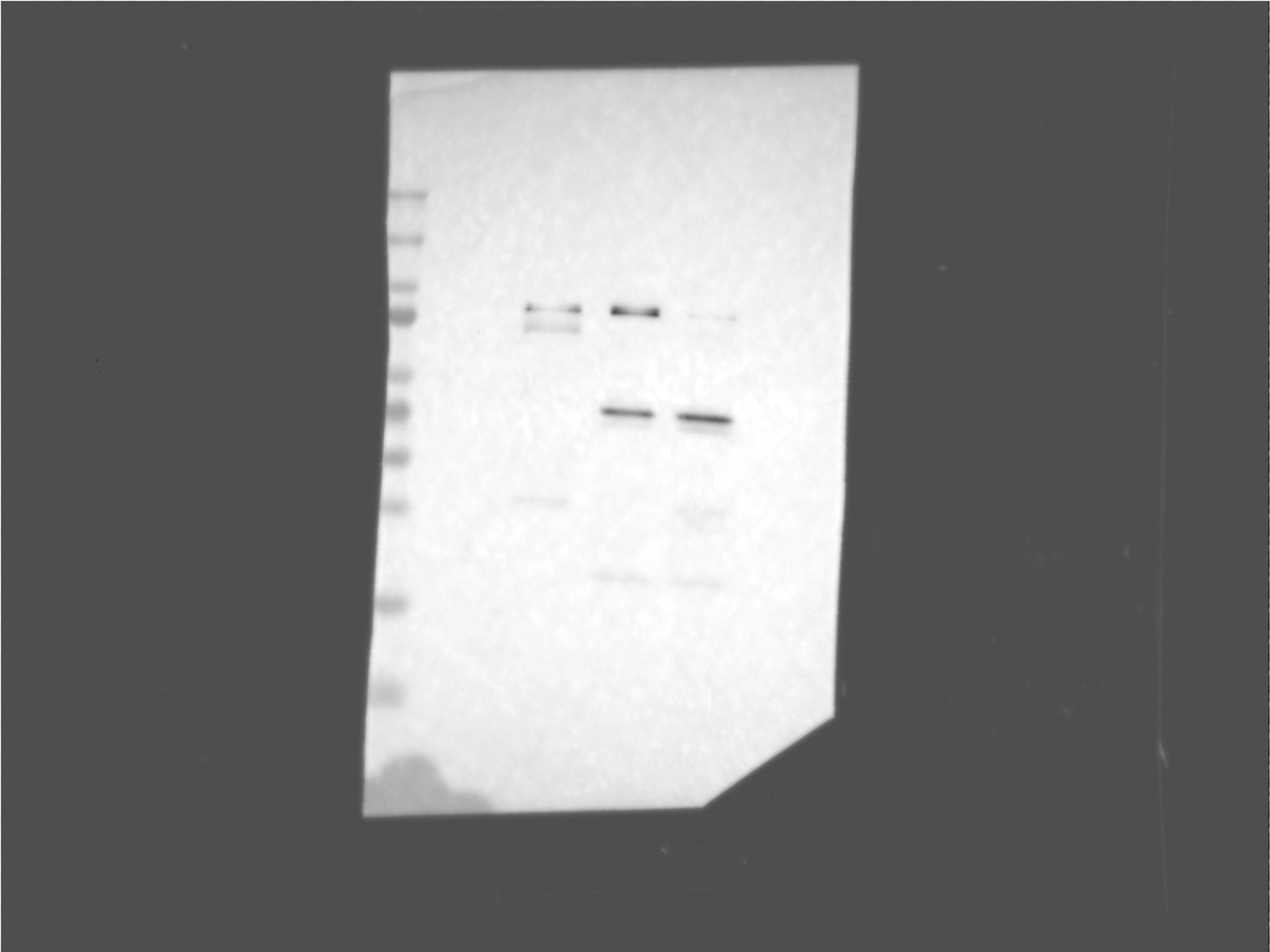

Supplement: Figure 2—figure supplement 5—source data 1. [file elife-95828-fig2-figsupp5-data1.zip › Figure 2-figure supplement 5-source data 1/anti-RFP.tif]

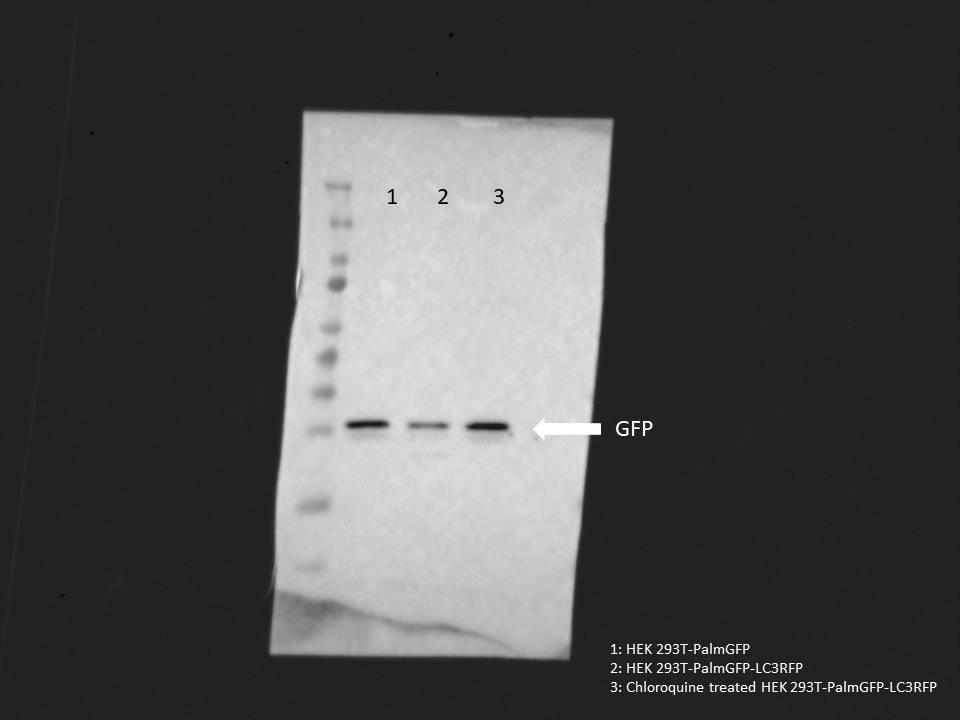

Supplement: Figure 2—figure supplement 5—source data 2. [file elife-95828-fig2-figsupp5-data2.zip › Figure 2-figure supplement 5-source data 2/anti-GFP.TIF]

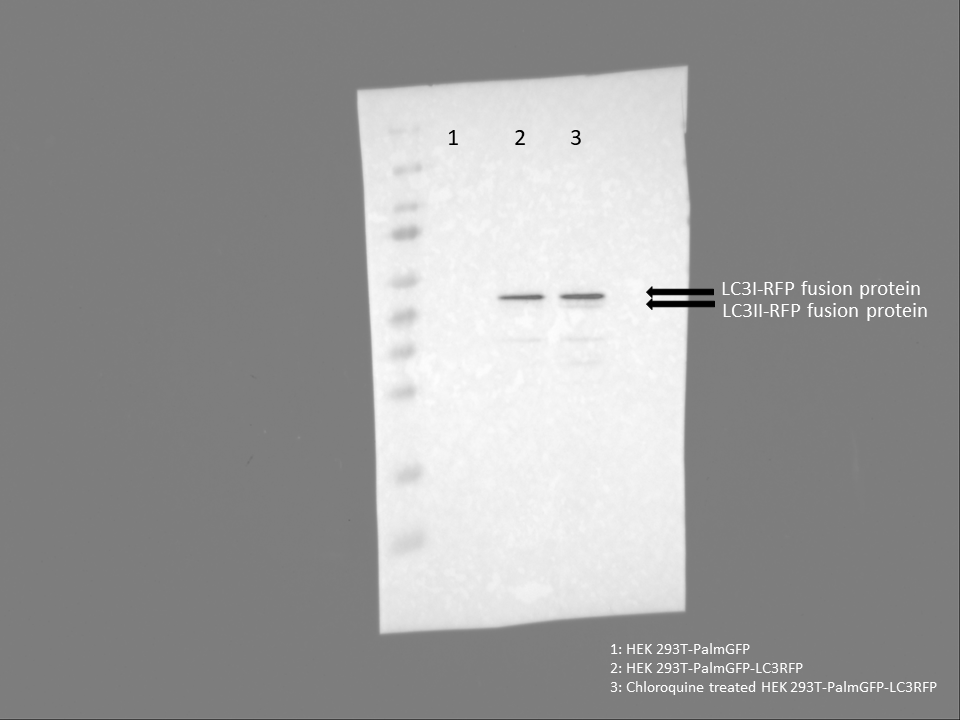

Supplement: Figure 2—figure supplement 5—source data 2. [file elife-95828-fig2-figsupp5-data2.zip › Figure 2-figure supplement 5-source data 2/anti-LC3A.TIF]

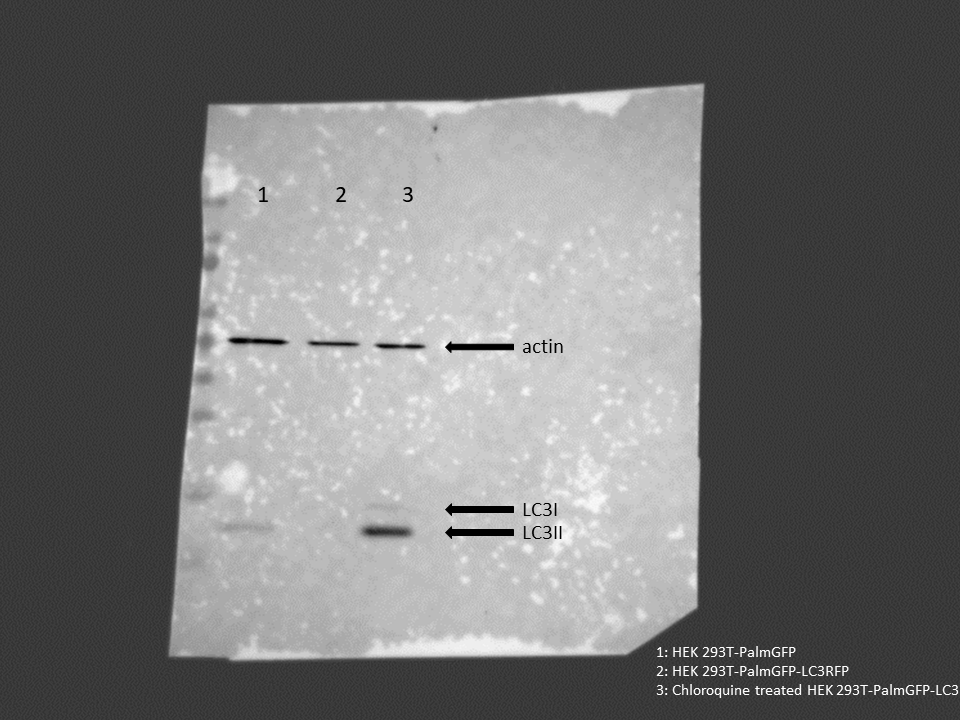

Supplement: Figure 2—figure supplement 5—source data 2. [file elife-95828-fig2-figsupp5-data2.zip › Figure 2-figure supplement 5-source data 2/anti-LC3B.TIF]

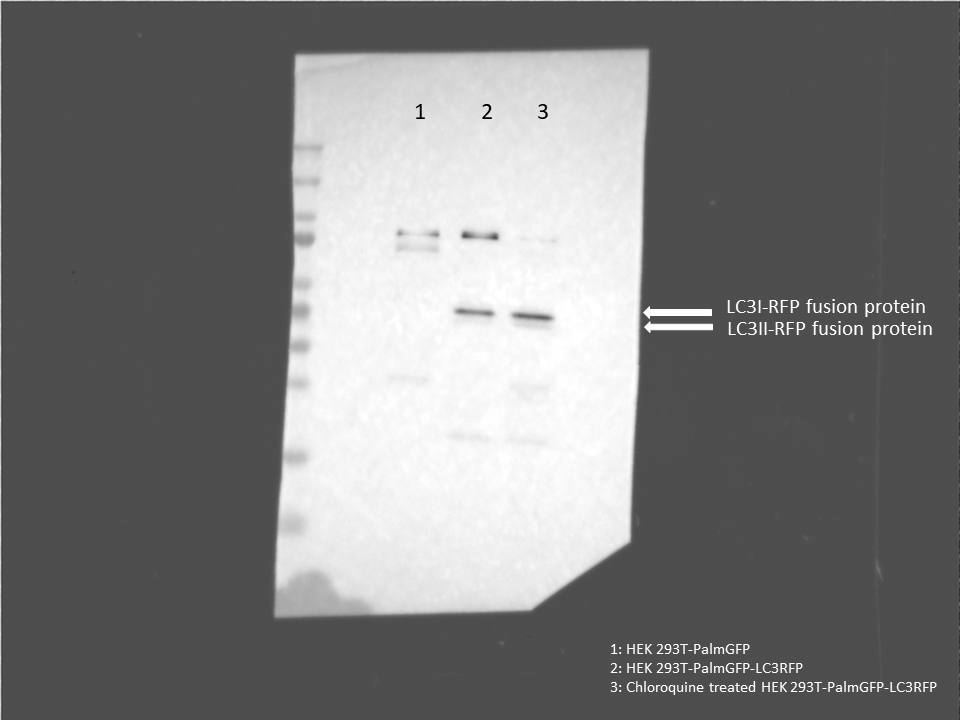

Supplement: Figure 2—figure supplement 5—source data 2. [file elife-95828-fig2-figsupp5-data2.zip › Figure 2-figure supplement 5-source data 2/anti-RFP.TIF]

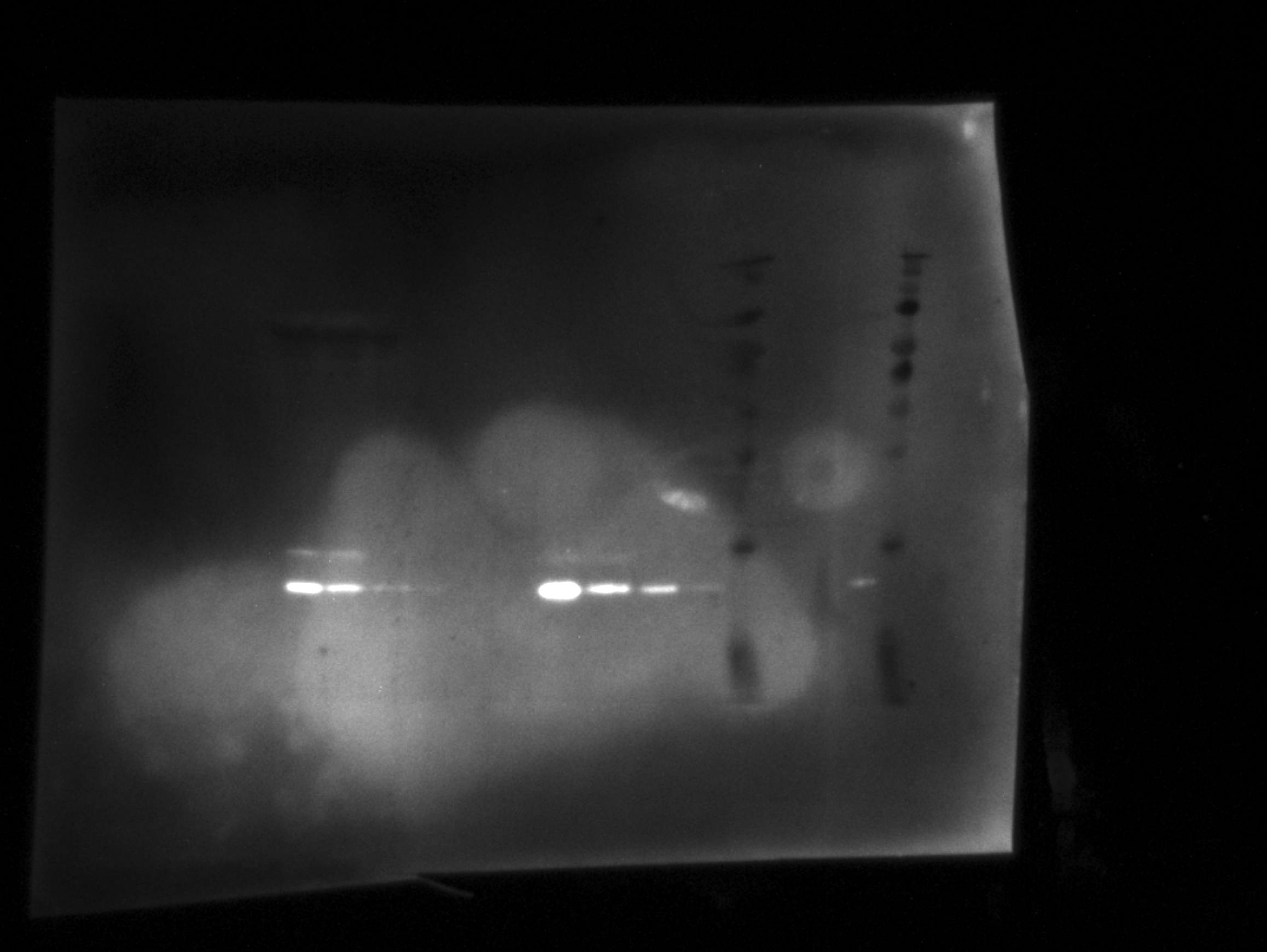

Supplement: Figure 3—source data 2. [file elife-95828-fig3-data2.zip › Figure 3-source data 3/anti-LC3B-1 inverted.tif]

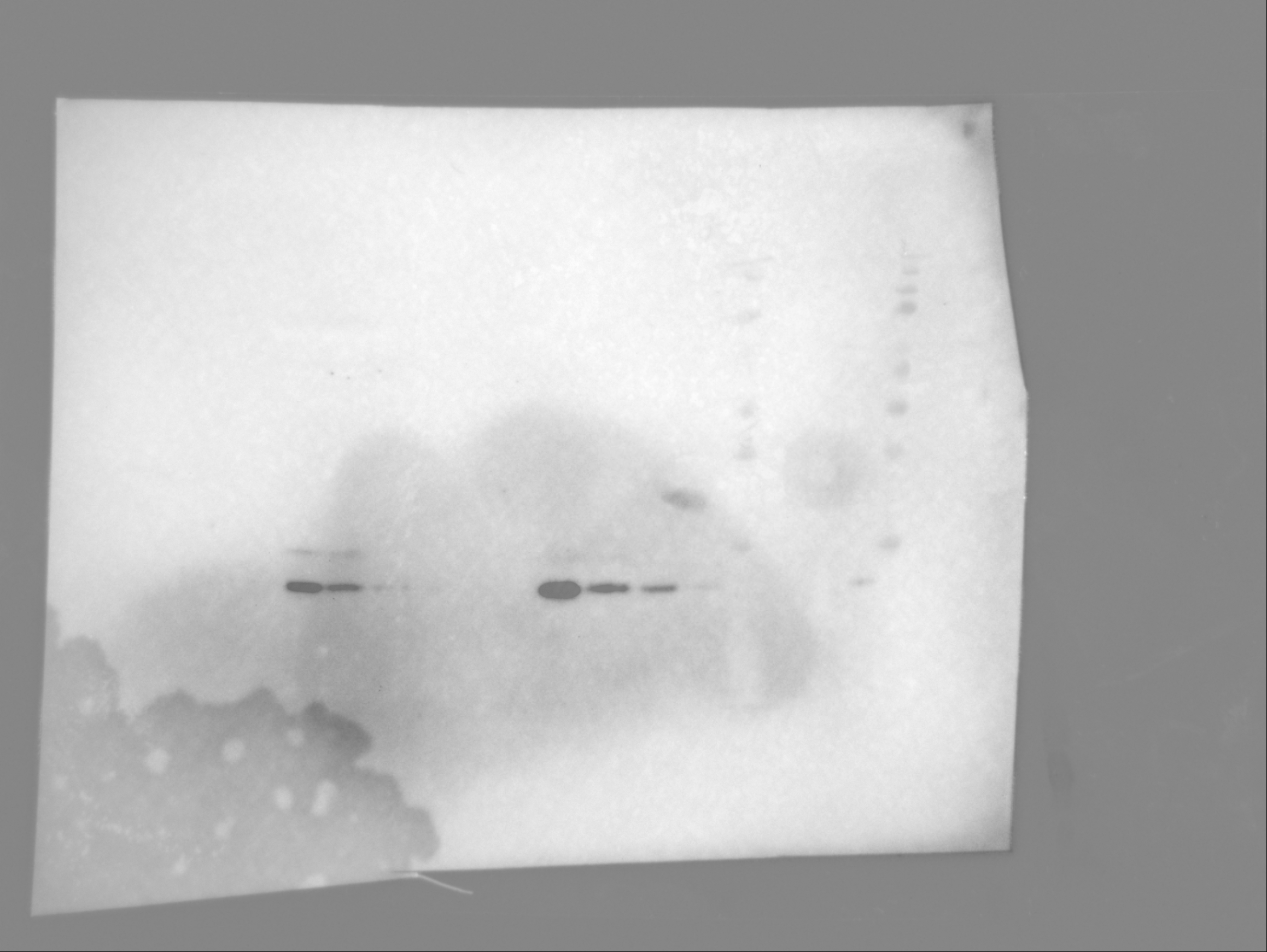

Supplement: Figure 3—source data 2. [file elife-95828-fig3-data2.zip › Figure 3-source data 3/anti-LC3B-1.tif]

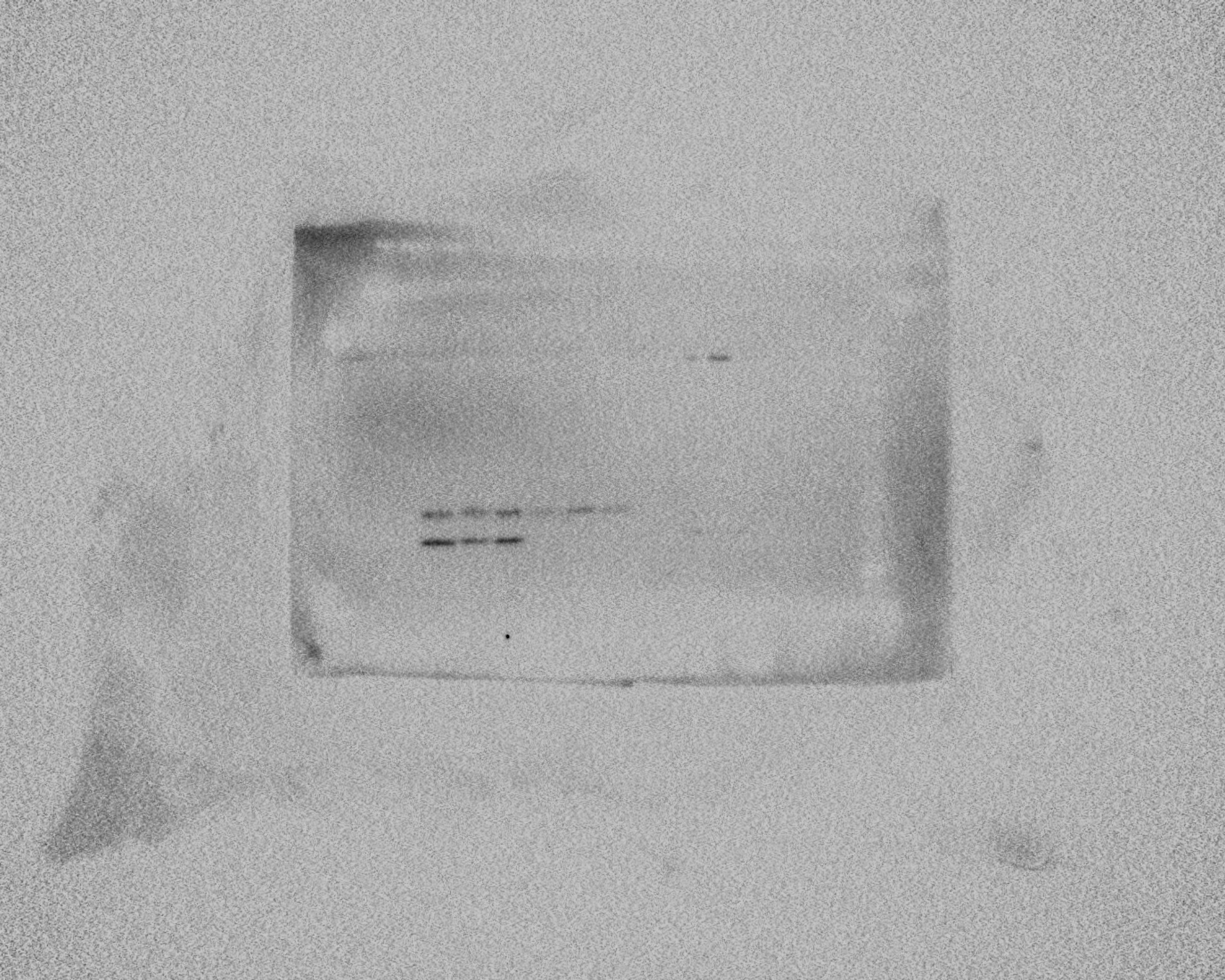

Supplement: Figure 3—source data 2. [file elife-95828-fig3-data2.zip › Figure 3-source data 3/anti-LC3B-2.tif]

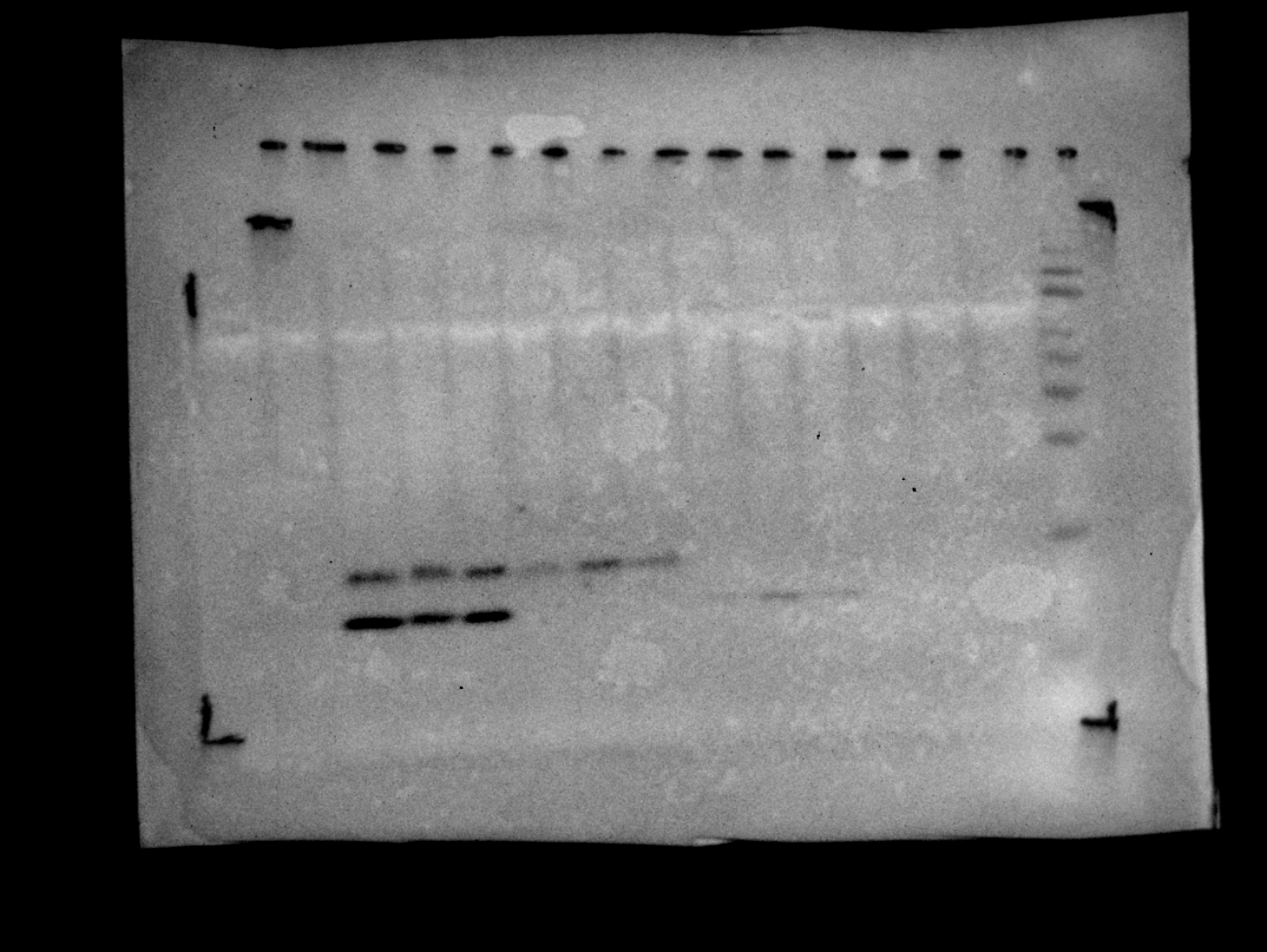

Supplement: Figure 3—source data 2. [file elife-95828-fig3-data2.zip › Figure 3-source data 3/anti-LC3B-3.tif]

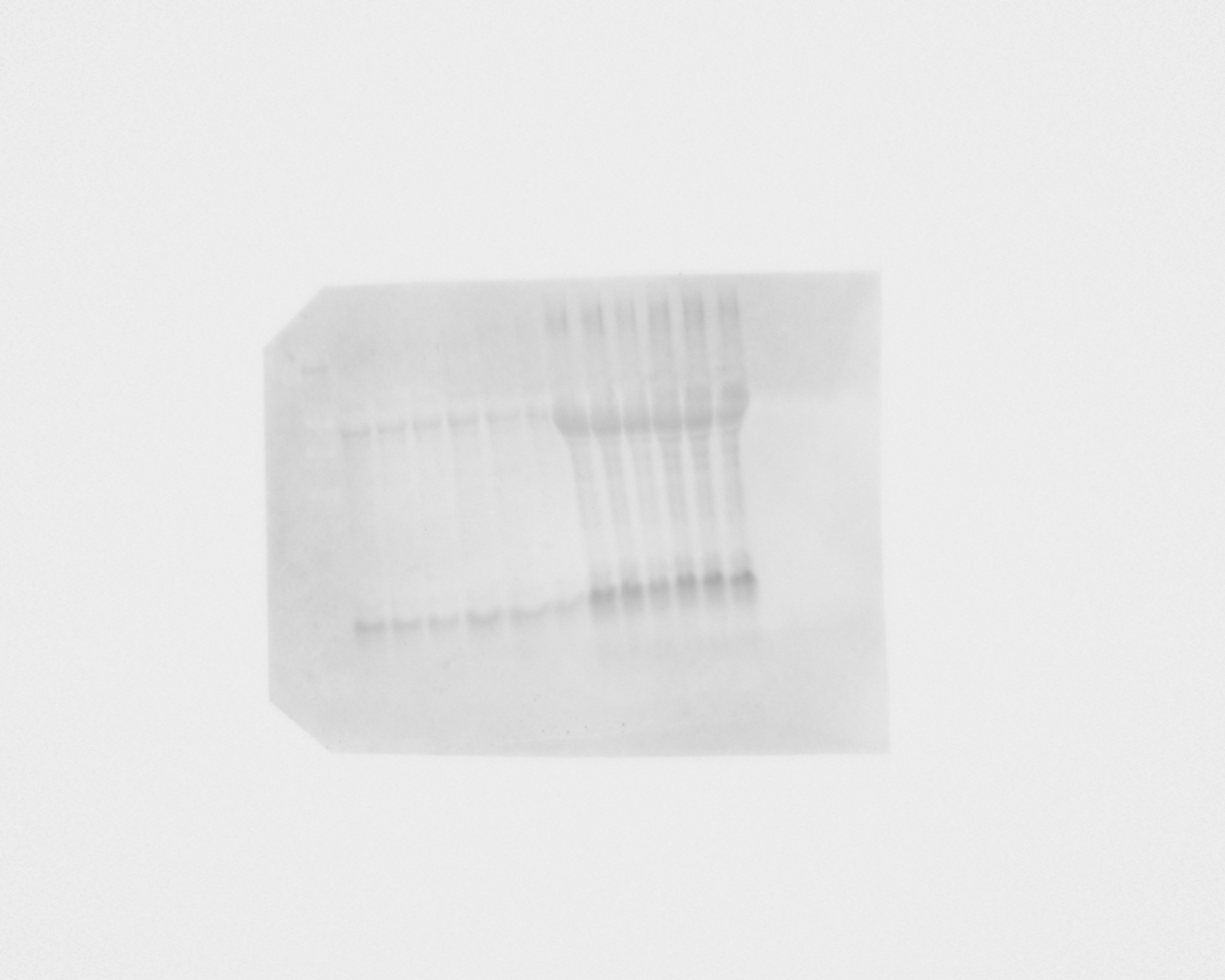

Supplement: Figure 3—source data 2. [file elife-95828-fig3-data2.zip › Figure 3-source data 3/anti-LC3B-4.tif]

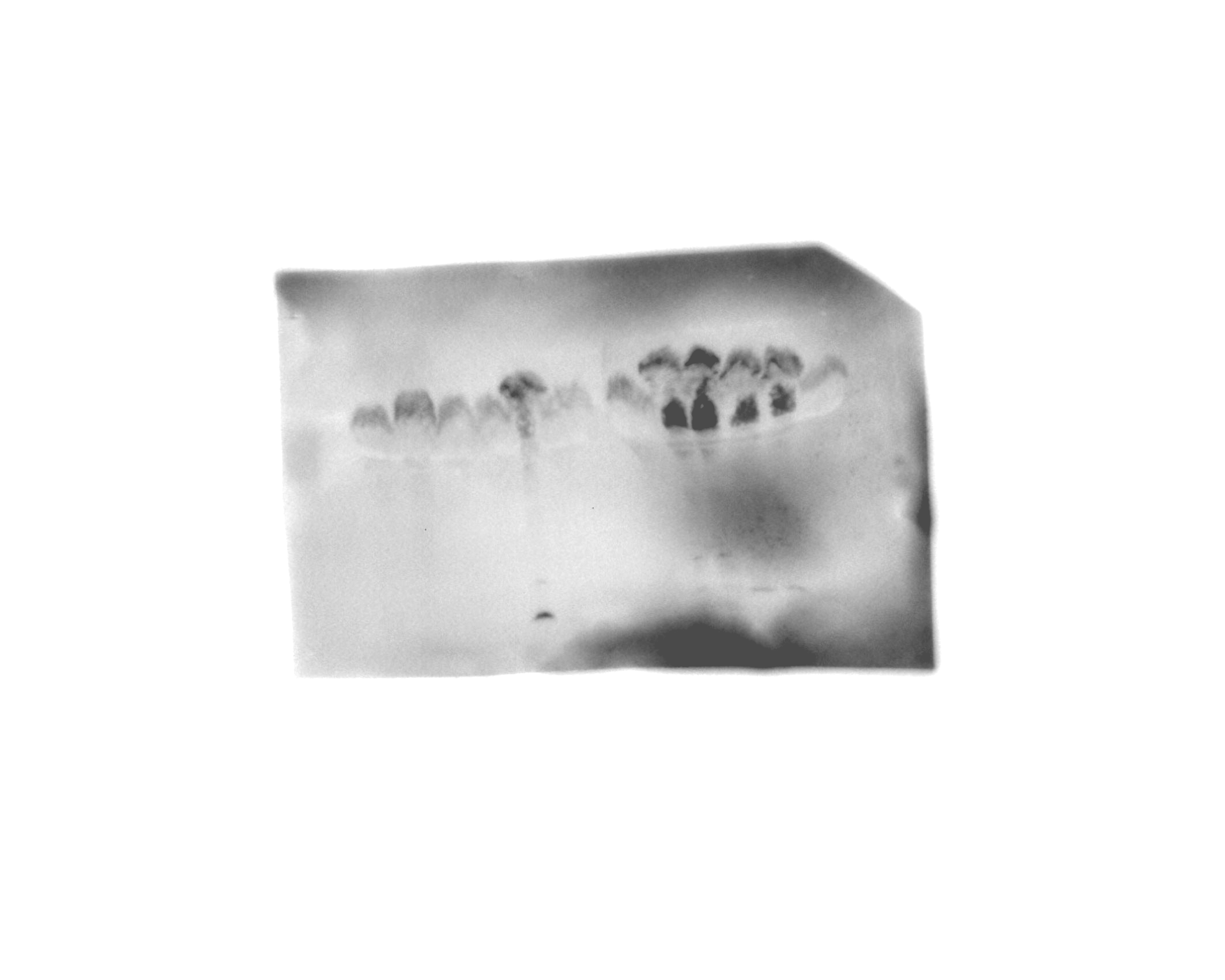

Supplement: Figure 3—source data 2. [file elife-95828-fig3-data2.zip › Figure 3-source data 3/anti-LC3B-5.tif]

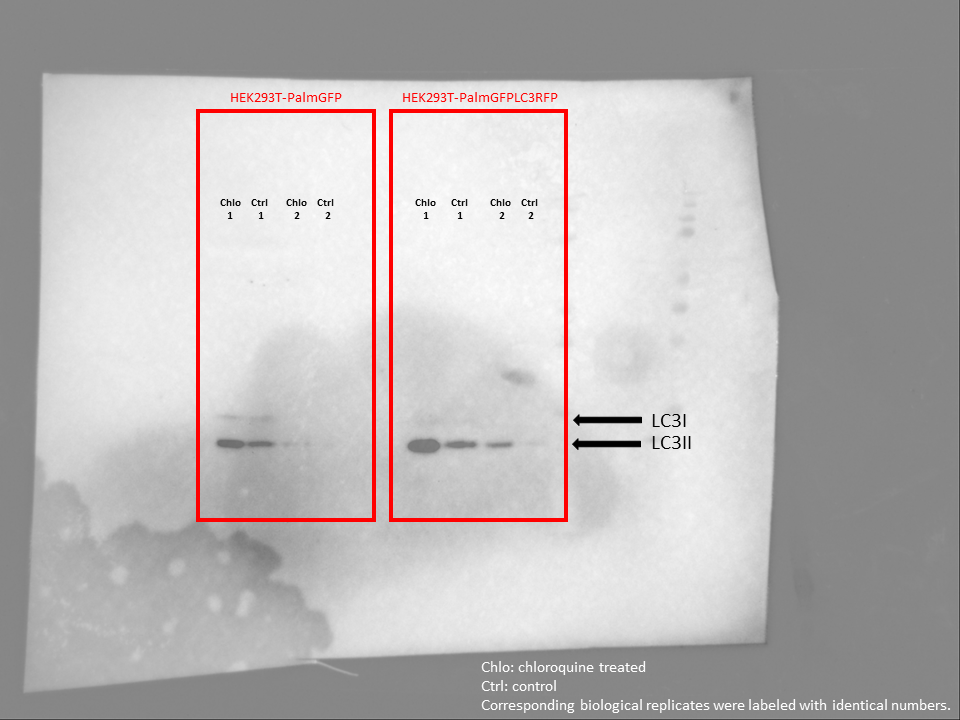

Supplement: Figure 3—source data 3. [file elife-95828-fig3-data3.zip › Figure 3-source data 4/anti-LC3B-1.TIF]

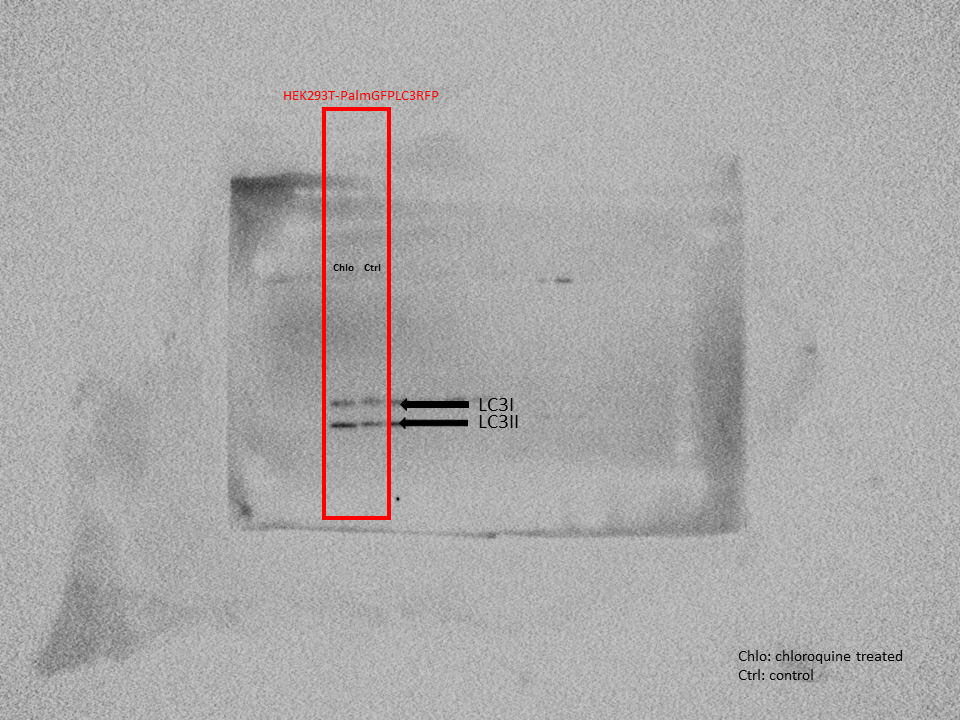

Supplement: Figure 3—source data 3. [file elife-95828-fig3-data3.zip › Figure 3-source data 4/anti-LC3B-2.TIF]

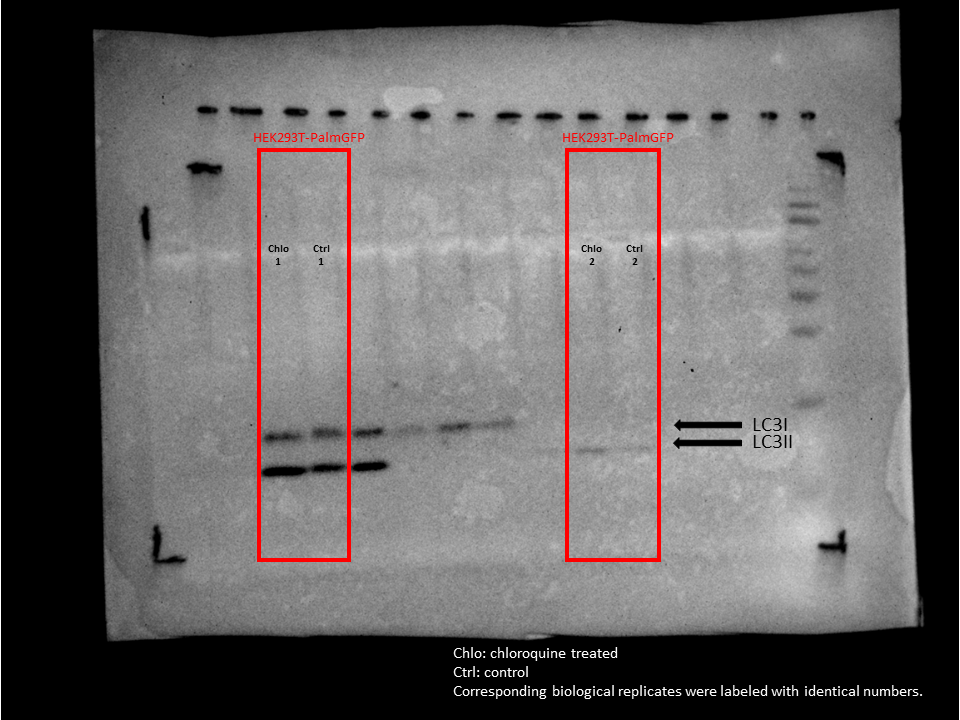

Supplement: Figure 3—source data 3. [file elife-95828-fig3-data3.zip › Figure 3-source data 4/anti-LC3B-3.TIF]

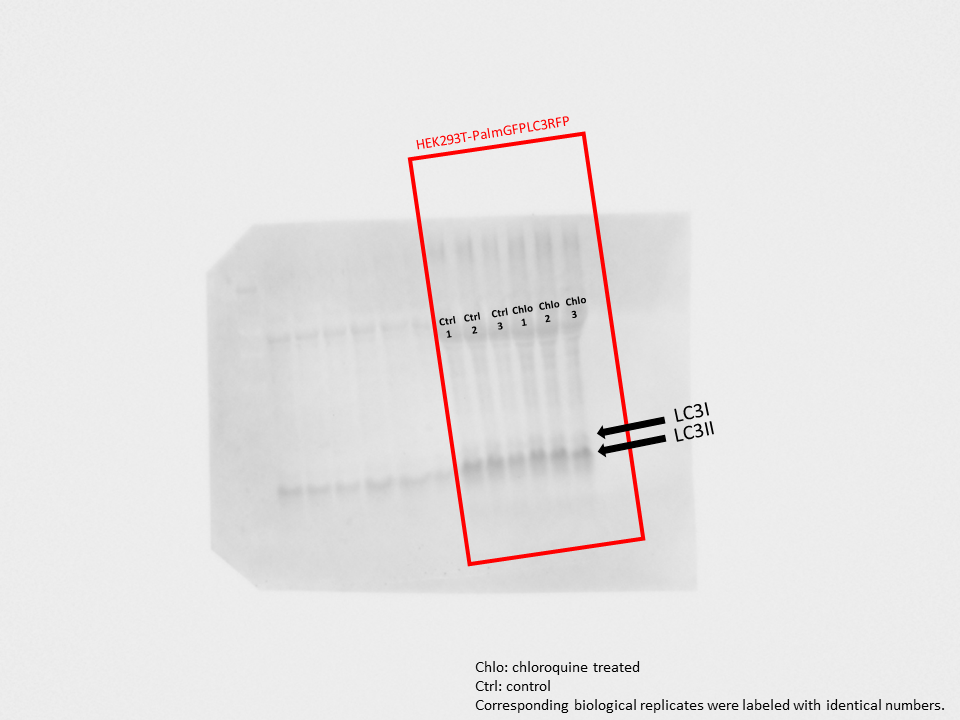

Supplement: Figure 3—source data 3. [file elife-95828-fig3-data3.zip › Figure 3-source data 4/anti-LC3B-4.TIF]

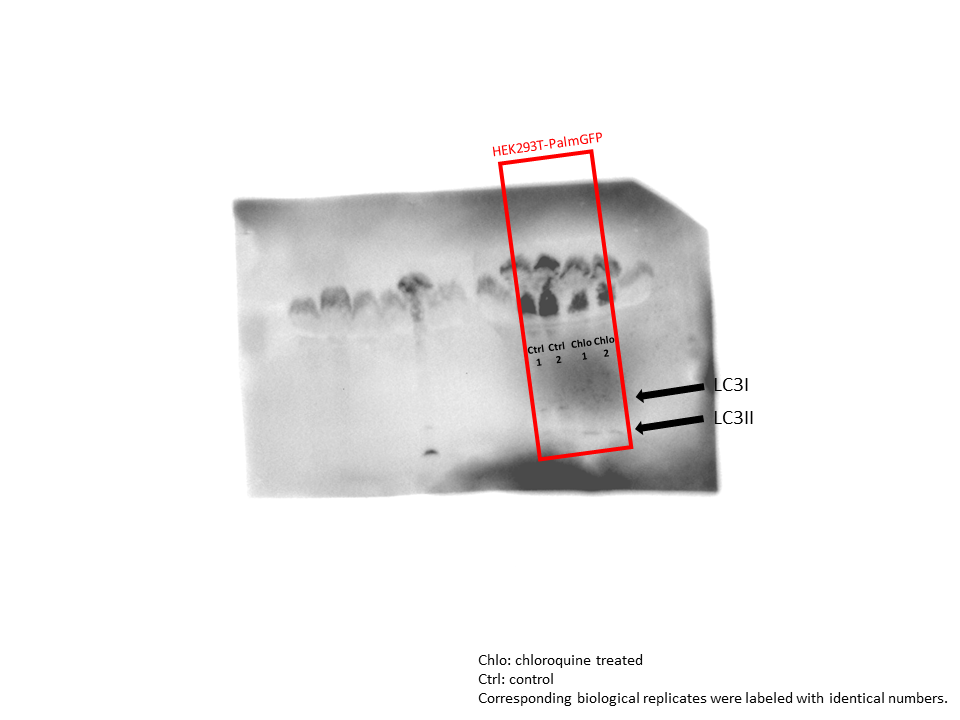

Supplement: Figure 3—source data 3. [file elife-95828-fig3-data3.zip › Figure 3-source data 4/anti-LC3B-5.TIF]
